# Mouse maternal systemic inflammation at the zygote stage causes blunted cytokine responsiveness in lipopolysaccharide-challenged adult offspring

**DOI:** 10.1186/1741-7007-9-49

**Published:** 2011-07-19

**Authors:** Charlotte L Williams, Jessica L Teeling, V Hugh Perry, Tom P Fleming

**Affiliations:** 1 School of Biological Sciences, University of Southampton, Mailpoint 840, Level D Laboratories & Pathology Block, Southampton General Hospital, Tremona Road, Southampton SO16 6YD, UK

## Abstract

**Background:**

The preimplantation embryo is sensitive to culture conditions *in vitro *and poor maternal diet *in vivo*. Such environmental perturbations can have long-lasting detrimental consequences for offspring health and physiology. However, early embryo susceptibility to other aspects of maternal health and their potential long-term influence into adulthood is relatively unexplored. In this study, we established an *in vivo *mouse model of maternal periconceptional systemic inflammation by intraperitoneal lipopolysaccharide (LPS) administration on the day of zygote formation and investigated the consequences into adulthood.

**Results:**

In the short term, maternal LPS challenge induced a transient and typical maternal sickness response (elevated serum proinflammatory cytokines and hypoactive behaviour). Maternal LPS challenge altered preimplantation embryo morphogenesis and cell lineage allocation, resulting in reduced blastocyst inner cell mass (ICM) cell number and a reduced ICM:trophectoderm cell ratio. In the long term, diverse aspects of offspring physiology were affected by maternal LPS treatment. Whilst birthweight, growth and adult blood pressure were unaltered, reduced activity in an open-field behaviour test, increased fat pad:body weight ratio and increased body mass index were observed in male, but not female, offspring. Most importantly, the maternal LPS challenge caused corticosterone-independent blunting of the serum proinflammatory cytokine response to innate immune challenge in both male and female offspring. The suppressed state of innate immunity in challenged offspring was dose-dependent with respect to the maternal LPS concentration administered.

**Conclusions:**

These results demonstrate for the first time that the preimplantation embryo *in vivo *is sensitive to maternal systemic inflammation, with effects on blastocyst cell lineage allocation and consequences for behaviour, adiposity and innate immune response in adult offspring. Critically, we identify a novel mechanism mediated through maternal-embryonic interactions that confers plasticity in the development of the innate immune system, which is potentially important in setting postnatal tolerance to environmental pathogens. Our study extends the concept of developmental programming of health and disease to include maternal health at the time of conception.

## Background

Substantial evidence supports the concept that adult health and physiology are influenced by environmental factors during the gestational and infant stages of development [1-3]. The developmental origins of health and disease (DOHaD) hypothesis proposes that such programming may be contributing to the increasing prevalence of hypertension, diabetes and other noncommunicable diseases in recent decades [1]. Programming can be initiated even during the periconceptional period: the preimplantation embryo has proven sensitive to environmental perturbation mediated through maternal diet or *in vitro *culture, with concomitant postnatal dysfunction of cardiovascular, metabolic and behavioural systems [2-6]. Whether other aspects of periconceptional maternal health (for example, infection, inflammation) can induce long-term programming has not yet been explored in detail.

Inflammation is associated with the release of potent cellular mediators (for example, cytokines, glucocorticoids (GCs)) which communicate at autocrine and paracrine levels. Such mediators alter and regulate homeostasis and may modify the developmental environment. Maternal infection and inflammation during mid- to late gestation affects offspring behaviour [7,8], immunity [9-11], metabolic status [12] and hypothalamic-pituitary-adrenal (HPA) axis functioning [13]. Little is known about the postnatal sequelae of maternal inflammation during the periconceptional period, although an association with an increased risk of schizophrenia has been reported [14,15]. In this study, we established an *in vivo *mouse model of maternal periconceptional systemic inflammation (MPSI) by lipopolysaccharide (LPS) administration on the day of zygote formation.

We found that the maternal LPS challenge induces a typical and transient sickness response and, critically, altered developmental programming with abnormal blastocyst cell lineage allocation and led to postnatal changes in behaviour, adiposity and, following innate immune challenge, in cytokine release. Our study therefore establishes that maternal sickness at the time of conception, or immune response conditions associated with it, can have lifelong consequences on offspring health, thereby extending the concept of DOHaD. Moreover, our data show for the first time that the responsiveness of the innate immune system in an adult mammal is subject to environmental conditions originating at the time of conception.

## Results

### Maternal inflammatory response

**Figure 1:**
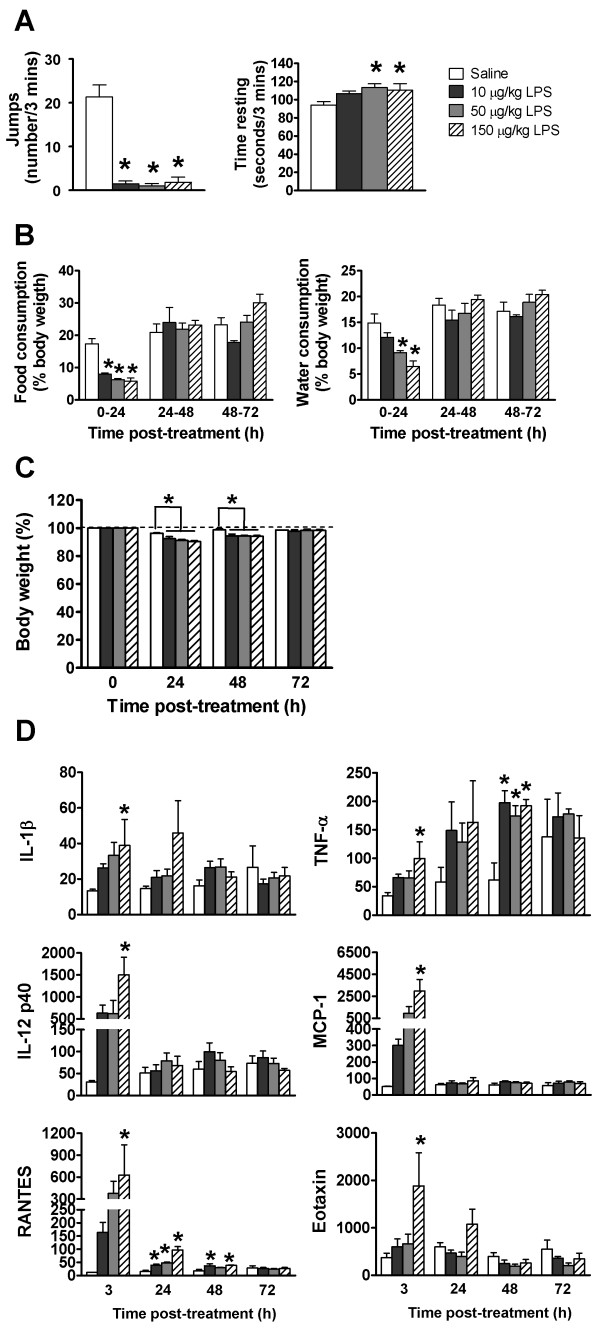
**Maternal inflammatory response following treatment with saline or LPS doses**. LPS treatment (particularly 50 and 150 μg/kg) **(A) **reduced jumping activity and increased time spent resting in an open-field test at one hour posttreatment, **(B) **reduced food and water consumption during the first 24 hours posttreatment, **(C) **reduced body weight at 24 and 48 hours posttreatment (dashed line = preinjection body weight) and **(D) **increased serum concentration (pg/mL) of inflammatory cytokines posttreatment. Maternal body weight at each 24-hour interval posttreatment is expressed as a percentage of pretreatment body weight, as were food and water consumption during each 24-hour period posttreatment. All values represent means ± standard error of the mean (SEM). *P ≤ 0.05 vs. control (*n *= 5 to 7 mothers per treatment).

Intraperitoneal administration of LPS (10, 50 or 150 μg/kg dose vs. control) into dams at gestational day (GD) 0.5 (zygote stage) induced a typical sickness response, including associated metabolic and behavioural changes (Figure 1). One hour after treatment LPS-injected mice displayed reduced open-field activity, including a 90% to 95% reduction in jumping activity (*P *< 0.001 for all doses) and increased time spent resting (*P *< 0.05 at 50 and 150 μg/kg LPS doses) (Figure 1A). At 24 hours post-LPS injection, food consumption was reduced at all doses (10% to 12% reduction; P < 0.001) (Figure 1B), as was water consumption at the 50 and 150 μg/kg LPS doses.

(*P *≤ 0.006) (Figure 1B). Thereafter food and water consumption were unchanged between treatment groups (Figure 1B). Body weight at 24 hours was reduced by 3.8% to 5.7% in an LPS dose-dependent manner (*P *< 0.001 at 50 and 150 μg/kg and P < 0.01 at 10 μg/kg LPS doses) (Figure 1C). Body weight was similarly reduced at 48 hours (4.2% to 4.5% reduction; *P *≤ 0.003) (Figure 1C) but returned to control levels by 72 hours (Figure 1C). A selection of the 23 cytokines detected by multiplex assay in maternal serum at 3 to 72 hours posttreatment is shown in Figure 1D, and the remaining cytokines are shown in Figure S1 in Additional file 1. Serum concentrations of most cytokines peaked at three hours post-LPS treatment, with the 150 μg/kg LPS dose resulting in a three- to fivefold increase in IL-1β, IL-3, IL-10, eotaxin, granulocyte-macrophage colony-stimulating factor (GM-CSF) and macrophage inflammatory protein 1α (MIP-1α; CCL3) (*P *< 0.05). Greater increases were seen at three hours in IL-12 p40 (48-fold; *P *= 0.001), monocyte chemoattractant protein 1 (MCP-1; CCL2) (58-fold; *P *= 0.004) and regulated upon activation, normal T-cell expressed and secreted (RANTES; CCL5) (52-fold; P = 0.044). At 48 hours, most cytokine levels from LPS-treated groups were similar to those of controls; however, some levels remained elevated (IL-6, IL-12 p70, RANTES and TNF-α; *P *≤ 0.05), whilst others were decreased (IL-2, IL-10 and IL-13; *P *≤ 0.05) (Figure 1D and Additional file 1, Figure S1).

### Embryo recovery and blastocyst cell number

**Figure 2:**
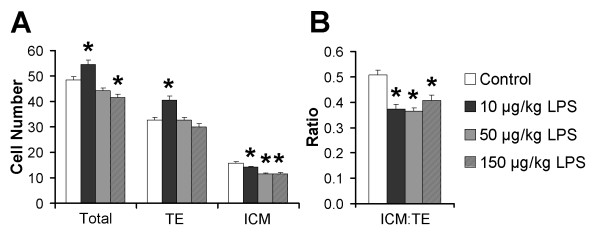
**GD 3.5 blastocysts from LPS-treated dams have fewer ICM cells and reduced ICM:TE cell ratios vs. controls**. **(A) **Mean total, TE and ICM cell numbers and **(B) **ICM:TE cell ratios. All values represent means ± SEM. *P ≤ 0.05 vs. control (*n *= 33 to 50 blastocysts per treatment; *n *= 4 to 8 mothers per treatment).

The effect of maternal LPS administration on preimplantation embryo development was assessed at GD 3.5. Maternal LPS had no effect on total number of embryos recovered or the proportion that were blastocysts (Table S1 in Additional file 2). However, blastocysts from all LPS treatments had reduced inner cell mass (ICM) cell numbers (14% reduced vs. control in the 10 μg/kg LPS group, *P *= 0.009; 33% reduced in the 50 and 150 μg/kg LPS groups, *P *< 0.001) (Figure 2A). Trophectoderm (TE) cell numbers were unaffected, except in the 10 μg/kg LPS group (21% increased, *P *< 0.001) (Figure 2A). Total blastocyst cell numbers were increased in the 10 μg/kg LPS group (*P *= 0.017) and reduced in the 150 μg/kg LPS group (*P *= 0.004) (Figure 2A). Such alterations in cell numbers resulted in reduced ICM:TE cell ratios in blastocysts from all LPS treatments (24% to 42% reduced, *P *≤ 0.001) (Figure 2B).

### Gestation, litter size and offspring growth

Gestation length, male:female ratio, mean birthweight and litter size were all unaffected, except for increased litter size in the 10 μg/kg LPS group (*P *= 0.046) (Table 1). The mean weekly body weights of male and female offspring from birth to 30 weeks were unaffected (Figure S2 in Additional file 3).

**Table 1:** Gestation and birth data for each maternal treatment group^a^

Maternal treatment (μg/kg LPS)	Number of mothers	Gestation length (days)	Total number of offspring	Mean litter size	Male:female ratio	Mean birthweight (g)
0 (saline control)	6	19	65	10.8 ± 0.6	1.1 ± 0.17	1.62 ± 0.02
10	6	19	82	13.7 ± 0.6*	1.4 ± 0.21	1.57 ± 0.01
50	6	19	76	12.7 ± 0.3	1.0 ± 0.16	1.57 ± 0.01
150	6	19	68	11.3 ± 1.0	1.1 ± 0.18	1.64 ± 0.02

^a^LPS: lipopolysaccharide. Values represent means ± SEM. *P ≤ 0.05 vs. control.

### Offspring behaviour

**Figure 3:**
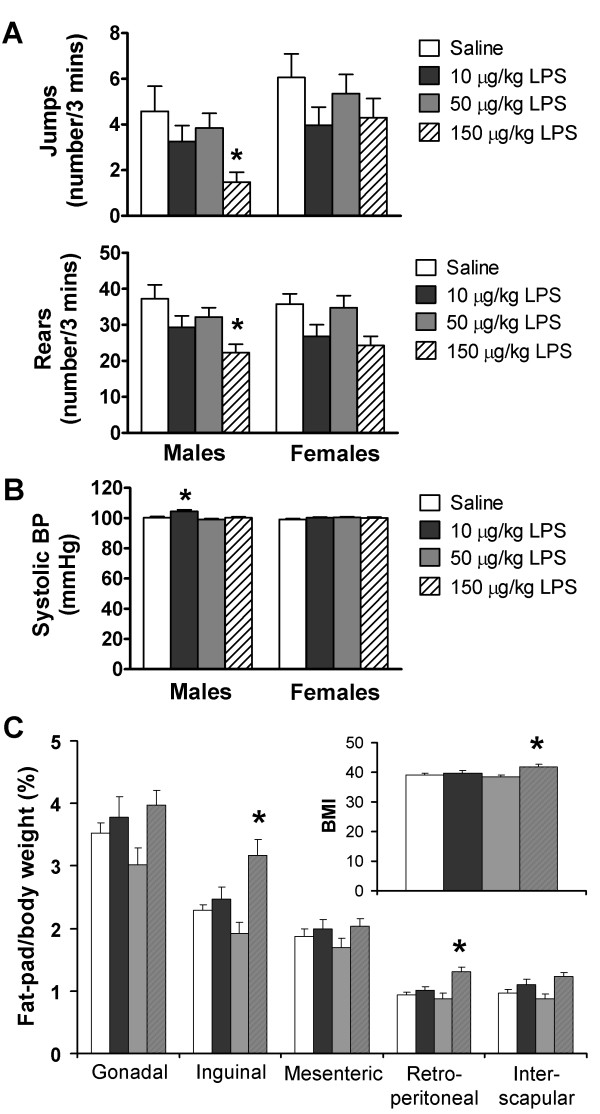
**MPSI caused dose-dependent, gender-specific abnormalities in adult offspring behaviour and adiposity, but no abnormalities in systolic blood pressure (SBP)**. **(A) **Reduced jumping and rearing open-field activity in male, but not female, offspring from 150 μg/kg LPS dams. **(B) **Increased SBP in male offspring from 10 μg/kg LPS dams. **(C) **Increased fat pad to body weight ratio (%) and BMI in male offspring from 150 μg/kg LPS dams. All values represent means ± SEM. *P ≤ 0.05 vs. control independent of maternal origin and litter size (*n *= 6 mothers per treatment; n = 18 male and 18 female offspring per treatment).

Integration of all weekly data (nine repeated analyses over weeks 4 to 23) into mean lifetime open-field activities showed that 150 μg/kg LPS males displayed reduced numbers of jumps and rears (reduced by 40% vs. control, *P *= 0.015, and reduced by 67% vs. control, *P *= 0.027, respectively) (Figure 3A), but no differences in distance travelled or time spent resting (Figure S3 in Additional file 4). Mean lifetime open-field activities of female offspring were unaffected (Figure 3A and Additional file 4, Figure S3). When open-field activities were examined on a weekly basis (Figure S4 in Additional file 5), male offspring from 150 μg/kg LPS-treated dams reared less than controls at weeks 4, 6, 11, 14 and 20 (all *P *< 0.05), while female offspring from 150 μg/kg LPS-treated dams reared less than controls only at week 8 (*P *= 0.001). In addition, male offspring from 150 μg/kg LPS-treated dams jumped less than controls at weeks 6 and 17 (*P *< 0.05), and rested longer at week 6 (*P *= 0.040). No differences between treatments were observed for females in number of jumps or time spent resting, and distance travelled was not different between treatments for males or females (Figure S4 in Additional file 5). Differences were independent of maternal origin and gestational litter size.

### Offspring systolic blood pressure

Integration of weekly measurements (three repeated analyses over weeks 16 to 28) into a 'lifetime' mean showed that systolic blood pressure (SBP) was not different, except in male offspring from 10 μg/kg LPS-treated dams, in which mean lifetime SPB was increased compared with controls (*P *= 0.029) (Figure 3B). On a weekly basis, male offspring from 10 μg/kg LPS-treated dams displayed increased SBP at weeks 16 and 28 compared with controls (*P *= 0.035 and *P *= 0.040, respectively) (Figure S5 in Additional file 6).

### Offspring body composition

Analysis of tissue:body weight ratios and body mass index (BMI) showed that compared with controls, male offspring from 150 μg/kg LPS-treated dams had a 28% increase in inguinal fat:body weight ratio (*P *= 0.031) (Figure 3C), a 29% increase in retroperitoneal fat:body weight ratio (*P *= 0.013) (Figure 3C) and a 7% increase in BMI (*P *= 0.033) (Figure 3C), but no differences in any organ:body weight ratio (Figures S6A and S6D in Additional file 7). In contrast, females showed no treatment group differences in fat pad:body weight ratio, BMI (Figure S6B in Additional file 7) or any organ:body weight ratio (Figure S6C in Additional file 7), except for liver:body weight ratio, which decreased by 12% in the 50 μg/kg LPS-treated dam group (*P *= 0.033) (Figure S6D in Additional file 7). All differences were independent of maternal origin and gestational litter size.

### Offspring serum cytokine profile

**Figure 4:**
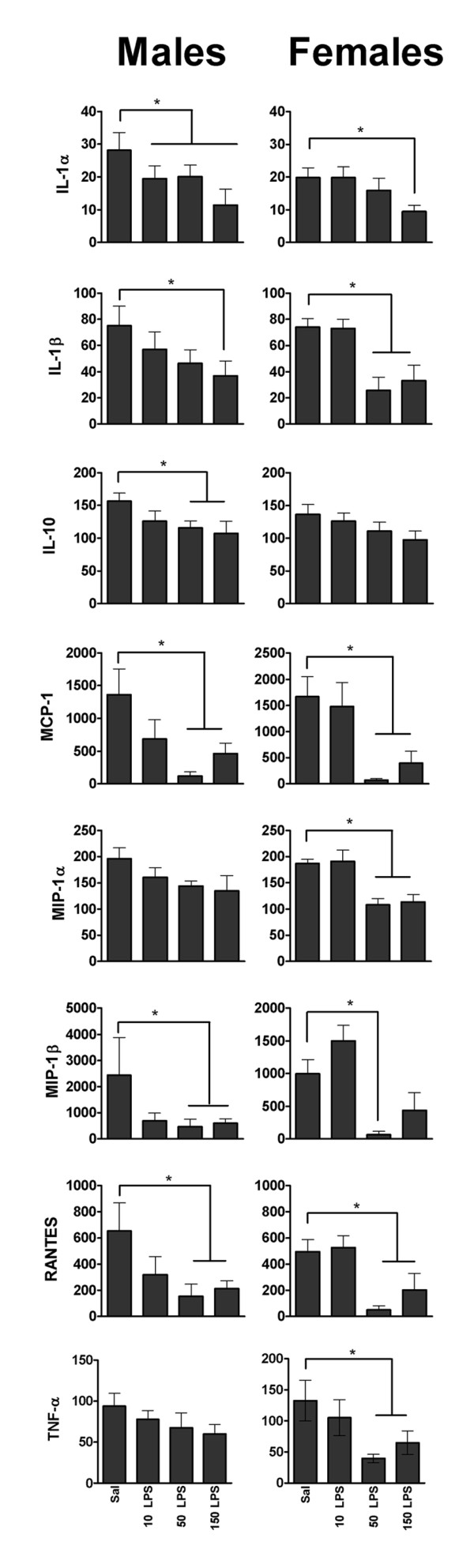
**Serum cytokine concentrations (pg/mL) of adult offspring from each maternal treatment group 3.5 hours after LPS challenge**. MPSI caused blunted release of predominantly innate-type cytokines in challenged offspring. A selection is shown here; the remaining cytokines are shown in Figure S3 in Additional file 4. All values represent means ± SEM. *P ≤ 0.05 vs. control independent of maternal origin and litter size (*n *= 6 mothers per treatment). Maternal treatments: Sal = saline; 10 LPS = 10 μg/kg LPS; 50 LPS = 50 μg/kg LPS; 150 LPS = 150 μg/kg LPS.

Offspring response to an innate immune challenge was assessed by measuring serum cytokine concentrations in LPS-injected (50 μg/kg) adult offspring of dams that were injected with saline or 10, 50 or 150 μg/kg LPS at the zygote stage. A selection of the 23 cytokines detected by the multiplex assay is shown in Figure 4. The remaining cytokines are shown in Figure S7 in Additional file 8. At 3.5 hours post-LPS injection, male and female offspring of LPS-treated dams had severely blunted serum levels of several cytokines compared with controls. Notably, the cytokine response of challenged offspring was either blunted in a maternal LPS dose-dependent fashion or not altered; in no case did maternal LPS treatment enhance offspring response. Challenged males from 10 μg/kg LPS-treated dams (10 LPS) had blunted levels of IL-1α (31% lower, *P *= 0.03) and granulocyte colony-stimulating factor (G-CSF) (99% lower, *P *= 0.042), while those from 50 μg/kg LPS-treated dams (50 LPS) had blunted levels of MCP-1 (91% lower, *P *= 0.003), IL-1α, IL-9, IL-10, G-CSF, MIP-1β (CCL4) and RANTES (26% to 91% lower, all *P *< 0.05) and those from 150 μg/kg LPS-treated dams (150 LPS) had blunted levels of IL-1α, IL-9 (44% to 60% lower, all *P *< 0.01), IL-1β, IL-10, MCP-1, MIP-1β and RANTES (31% to 75% lower, all *P *< 0.05). Challenged females from 10 μg/kg LPS-treated dams had a serum cytokine profile similar to that of controls. In contrast, challenged females from 50 μg/kg LPS-treated dams had blunted levels of IL-1β, IL-12 p40, G-CSF, keratinocyte-derived chemokine (KC; CXCL1), MCP-1, MIP-1α, MIP-1β, RANTES and TNF-α (42% to 99% lower, all *P *< 0.01) and those from 150 μg/kg LPS-treated dams had blunted levels of IL-1β, IL-12 p40, G-CSF, KC, MCP-1, MIP-1α (40% to 99% lower, all *P *< 0.01), IL-1α, IL-2, RANTES and TNF-α (51% to 59% lower, all *P *< 0.05).

### Offspring serum corticosterone

In addition to serum cytokines, serum corticosterone concentration was quantified in LPS-injected offspring from each maternal treatment group (the same groups described above in 'Offspring serum cytokine profile'). Serum corticosterone concentration of LPS-injected offspring was unaffected by maternal treatment (Figure S8 in Additional file 9).

## Discussion

We have established an *in vivo *mouse model of MPSI by intraperitoneal administration of increasing doses of LPS on the day of zygote formation (GD 0.5) and found developmental programming to be significantly altered. We report that MPSI changes blastocyst cellular organisation and induces a complex postnatal phenotype affecting adult behaviour, adiposity and response to an innate immune challenge in a gender-specific manner that is dependent upon the originating maternal LPS dose. These findings provide new insight into both the breadth of environmental cues that early embryos are sensitive to and the range of long-term adult physiological systems that are modulated as a consequence.

### Maternal response to LPS

Initially, we confirmed induction of an inflammatory response in LPS-injected dams. LPS treatment caused metabolic and behavioural changes typically associated with an inflammatory response, including reduced locomotor and exploratory activity, reduced food consumption and body weight, and increased circulating cytokine and chemokine levels. Long-term changes to offspring phenotype are known to result from a periconceptional maternal low-protein diet [2,16,17]. It could therefore be argued that the indirect effect of reduced food consumption might be the critical inductive event changing the adult phenotype. We believe this not to be the case, since (1) maternal food consumption was transiently reduced by an equivalent amount in all LPS groups, whilst changes in offspring phenotype were LPS dose-dependent; (2) reduction in maternal food consumption was temporally equivalent in all LPS groups, that is, maternal food consumption in all LPS groups returned to control levels at an equivalent rate; (3) maternal serum cytokine levels, a more direct measure of the inflammatory response, were LPS dose-dependent, corresponding with the resultant changes in offspring phenotype; and (4) maternal diet-induced programming is distinct from that reported here and affects particularly postnatal growth and cardiovascular function [16].

### Maternal LPS effect on blastocysts

Administration of LPS, including the low dose, on the day of zygote formation caused a reduction in GD 3.5 blastocyst ICM cell numbers and ICM:TE cell ratios. Reduced rodent blastocyst cell numbers have been induced by maternal protein undernutrition and *in vitro *culture and are associated with disease-related changes in postnatal physiology [2,6]. Similarly, mouse blastocyst cell numbers have been shown to be altered in response to maternal nutrition over a wider range [18]. Our current findings thus support the concept that altered blastocyst proliferation and lineage allocation may be useful early markers for diverse forms of developmental programming whetherlater effects on foetal and postnatal growth are induced (maternal protein restriction [16]) or not (current study). We also observed increased TE cell numbers coinciding with increased litter size only in the 10 μg/kg LPS group. This may reflect a 'trophic' effect mediated by a moderate release of inflammatory mediators, since cytokines and related signalling molecules are also important factors that are active during blastocyst development and implantation [19]. A greater degree of intrauterine growth restriction may have been exerted by the larger litter size in this low-dose LPS group and may explain the raised SBP (male offspring) seen here.

### Maternal LPS effects on offspring phenotype

Our most important finding is that MPSI results in long-term, gender-specific changes to offspring phenotype that are associated with a clear maternal LPS dose dependency. Male offspring from dams treated with the highest LPS dose displayed reduced locomotor and exploratory open-field behaviour, increased mass of specific fat pads, increased BMI and a blunted serum cytokine response to innate (LPS) immune challenge. Male offspring of the dams injected with the lower LPS doses (10 and 50 μg/kg) were much less affected, as were female offspring, which only showed the blunted serum cytokine response to LPS challenge. Notably, but not statistically significantly, the locomotor and exploratory behaviour of female offspring followed the same pattern as that of the males, suggesting that female behaviour is affected by MPSI, but to a lesser extent, such that programming of offspring behaviour may be graded rather than dictated by gender. Gender-specific differences in offspring phenotype have commonly been observed in DOHaD-related studies [2,6,16,17,20], but the underlying mechanisms are not understood yet. Interestingly, gender differences are evident in the preimplantation embryo from conception. For example, male embryos develop more rapidly than female embryos [21,22] and consequently may have different sensitivities to distinct environmental conditions that could thus contribute to the breadth of the postnatal outcomes discussed in this section}.

The combined postnatal phenotype, observed especially in male offspring of mothers that were injected with the high LPS dose, of reduced locomotor activity, increased adiposity and blunted cytokine response to LPS challenge may represent diverse manifestations from one or more causative pathways. The open-field behavioural assay has been effective in identifying broader conditions of ill health in mice, including cardiovascular and metabolic dysfunction [16,17], but provides symptomatic information rather than mechanistic insight. The adiposity and BMI outcomes are similar to those found in an earlier study where 790 μg/kg LPS administration to rat dams at midgestation caused central obesity, metabolic syndrome, hyperphagia and altered HPA functionality, but no change in blood pressure or lean body mass, in predominantly male offspring [12]. A similar offspring phenotype can result from administration of key inflammatory (IL-6 and TNF-α) or anti-inflammatory (dexamethasone) mediators during midgestation [23].

### Maternal LPS effect on offspring innate immunity

A novel finding of our study is that MPSI resulted in adult offspring with a severely blunted serum cytokine response to innate (LPS) immune challenge. The blunting was most pronounced in offspring of dams treated with the highest LPS doses, and, unlike most phenotypic changes in this study, it affected both male and female offspring. This blunted response was prominent in cytokines and chemokines (for example, IL-1β and MCP-1) typically produced by cells of the monocyte lineage (for example, macrophages), whereas cytokines of the lymphocyte lineage (for example, IL-3 and IL-5) were less affected. Interestingly, Shanks *et al*. [24] showed that exposure of neonatal rats to low-dose LPS protected them from adjuvant-induced arthritis in later life, suggesting a blunted response to infection and inflammation, in accordance with the findings of the present study.

Physiologically, the inflammatory response is controlled at a number of levels in a context- and cell-specific manner [25]; however, major mechanisms include immunosuppressive signalling via GC pathways, peroxisome proliferator-activated receptors (PPARs), regulatory T cells (T_regs_), alterations in Toll-like receptor (TLR) sensing and/or signalling pathways and epigenetic modifications [25-27]. Interestingly, altered expression of GC receptors (GRs) and PPARs has been found in offspring born to mothers fed a protein-restricted diet [28,29], and increased T_regs _have been found in cord blood from neonates born to farm-exposed mothers [30]. Thus, several pathways with the potential to regulate inflammation are known to be susceptible to programming influences. In the present study, production of the major rodent GC, corticosterone, was unaffected by MPSI, indicating that the blunted offspring cytokine response was not due to altered GC production. Furthermore, GCs possess broad immunosuppressive activity, whilst the blunting observed in this study predominantly affects innate cytokines. Taken together, these data support a mechanism that affects innate immune cells specifically, for example, altered TLR signalling and/or epigenetic modifications to cytokine promoters. Other aspects of offspring immune status await investigation.

The blunted cytokine response to LPS challenge is reminiscent of the endotoxin tolerance (ET) phenomenon, wherein transient hyporesponsiveness to endotoxins (LPS) follows an initial sublethal exposure [31]. However, the long-lasting intergenerational phenotype of MPSI is distinct from ET. Crucially, ET is not a state of total unresponsiveness, because LPS signalling in endotoxin-tolerant cells induces antimicrobial effector functions without concomitant expression of inflammatory cytokines [25]. In this way, the host is protected from a deleterious and potentially fatal cytokine storm (increased tolerance) whilst maintaining antipathogen defences (sustained resistance). In animals, host tolerance has been studied far less extensively than resistance; however, the importance of tolerance strategies for host survival and host-pathogen interactions is becoming increasingly known [32-34]. At a physiological level, we therefore view the current findings as evidence for a novel form of adaptive response [1] whereby maternal experience can lead to an increased level of postnatal tolerance which, within an anticipated pathogen-rich environment, would protect the offspring from the cytotoxic effects of their own frequent inflammatory responses. Cytokine hyporesponsiveness in ET is thought to be regulated at least in part by epigenetic mechanisms [31], just as metabolic responses are evident in diet-mediated developmental programming [28,29]. The molecular mechanisms underlying the blunted cytokine response in the current intergenerational model will be the subject of future research.

## Conclusions

In summary, we have identified for the first time that a maternal periconceptional inflammatory response acts as a mediator of developmental programming to preimplantation embryos. This environmental cue not only alters the cellular organisation of the blastocyst but also leads to permanent, gender-specific changes in adult offspring behaviour, adiposity and innate immune reactivity. Adult offspring are hyporesponsive to inflammatory stimuli which may confer host protection from inflammation-associated tissue damage. These findings in an animal model extend the repertoire of environmental conditions that preimplantation embryos are sensitive to and substantiate the need for broader analysis of underlying molecular mechanisms and applicability across species, including humans.

## Methods

### Animal treatments

With UK Home Office licence and local ethics committee approval, MF1 mice were bred in-house at the University of Southampton Biomedical Research Facility and kept on a 0700 to 1900 hours light cycle with *ad libitum *access to standard rodent chow. Virgin females (7 to 8.5 weeks old and 27 to 33 g in weight) were naturally mated overnight with MF1 studs. The following morning plug-positive females were housed individually and randomly assigned to receive either sterile saline (0.85%) or one of the following three LPS (*Salmonella enterica *serovar Enteritidis; Sigma, St Louis, MO, USA) dosages: 10, 50 or 150 μg/kg body weight (freshly prepared on each occasion). Saline or LPS was administered by intraperitoneal injection of 100-μL volumes between 1400 and 1600 hours on the day of plug (that is, at the zygote stage, GD 0.5). Mice were returned to their cages following treatment and were housed individually until required for experiments.

### Maternal inflammatory response

A group of mated females were assessed to characterise maternal response to saline (control) or LPS dosages. A subset from each treatment group were assessed for sickness-related behaviour and body weight. At one hour posttreatment, mice underwent an open-field test (see below in Offspring Analyses: Behaviour) to assess locomotor activity. Treated females and their chow and water bottles were weighed at 0, 24, 48 and 72 hours posttreatment to assess body weight and consumption of food and water. Another subset from each treatment group were killed by cervical dislocation at 3, 24, 48 and 72 hours posttreatment, maternal blood was collected by cardiac puncture and serum was aliquoted and stored at -80°C. Maternal serum cytokine profiles were assessed by using the Bio-Plex Pro Mouse Cytokine 23-plex panel (Bio-Rad Laboratories, Hercules, CA, USA).

### Embryo recovery and differential nuclear labelling

A group of females from each treatment group were killed as described above in Maternal inflammatory response) at GD 3.5, and embryos were flushed from uteri using prewarmed (37°C) H6 medium with 4 mg/mL BSA. Freshly collected blastocysts were analysed for cell numbers within the TE and ICM lineages by differential nuclear labelling as described previously [35] with modifications [2]. Blastocysts were viewed and imaged using a Zeiss Axiovert 200 fluorescence microscope (Carl Zeiss Ltd, Welwyn Garden City, Hertfordshire, UK) and MetaMorph version 6.2r6 software (Universal Imaging Corp.).

### Offspring analyses

For assessment of postnatal phenotype, females from each treatment group were bred to term. At birth, litters were standardised to six (three median weight offspring of each gender) and each offspring was assigned a neutral code that did not reflect the treatment group to which it belonged. Mice were identified by their codes throughout the study, and the results were not decoded until data analysis was performed at the end of the study. Offspring were weaned at three weeks with littermates of the same gender housed together.

#### Growth

Offspring were weighed weekly from birth to 30 weeks of age.

#### Behaviour

Open-field tests were conducted at 4, 5, 6, 8, 11, 14, 17, 20 and 23 weeks. Open-field tests were conducted in the afternoon in a normally lit room, and, prior to the test, mice were habituated to the behaviour room for 30 minutes. The open-field arena consisted of a white acrylic base enclosed on four sides with transparent 0.7-cm-thick acrylic (dimensions 27.5 × 27.5 × 20 cm, model ENV-520; Med Associates Inc., Georgia, VT, USA). Laser beams crossing the open-field arena allowed animal movement to be detected and recorded automatically. Each open-field arena was surrounded by an aluminium shield that prevented the mouse under investigation from seeing other rodents. Data were acquired by using Activity Monitor version 4.0 software (Med Associates Inc.) for a three-minute period which started as soon as a mouse was detected in the arena [36]. Each mouse was individually placed in the centre of the open-field arena, and the distance travelled, time spent resting, number of jumps and number of rears was recorded by the computer. After each three-minute recording period, the mouse was returned to its home cage and the open-field arena was cleared of any droppings and wiped out with disinfectant solution and paper towels.

#### Systolic blood pressure

SBP was measured at 16, 22 and 28 weeks of age by tail cuff plethysmography [6] using an ITC model 229 blood pressure monitor (Linton Instruments, Norfolk, UK). SBP was taken at room temperatures of 27°C to 30°C, to which we allowed the mice to acclimatise themselves for 60 minutes prior to measurement. This practice encourages vasodilatation, thus facilitating detection of SBP. Mice were restrained in a ventilated acrylic tube, and their tails were threaded through the tail cuff. Each mouse was allowed to become acclimatised to the apparatus for five minutes prior to readings' being taken. At each time point, five sequential SBP readings were taken for each mouse, and the mean value of the three median readings was calculated and recorded.

#### Terminal treatments, blood collection and body composition

Offspring were killed by cervical dislocation at 40 weeks of age. Three and one-half hours before they were killed, a subset of male and female littermates were injected with 50 μg/kg LPS (as described above in Animal treatments). Blood was collected by cardiac puncture, then major organs (heart, lungs, liver, brain and left and right kidneys) and fat pads (interscapular, retroperitoneal, mesenteric, gonadal and inguinal) were dissected and weighed. Serum was extracted from blood samples and stored at -80°C.

#### Serum cytokine profiles

Offspring (and maternal, see above in Maternal inflammatory response{) serum cytokine profiles were assessed using the Bio-Plex Pro Mouse Cytokine 23-plex panel (Bio-Rad Laboratories) as per the manufacturer's instructions. Before analysis, thawed serum samples were diluted 1:4 using Bio-Plex mouse serum diluent (Bio-Rad Laboratories) as recommended by the manufacturer. Prepared plates were run on a Luminex 100 instrument (Luminex Corp., Austin, TX, USA) equipped with STarStation version 2.0 software (Applied Cytometry, Sheffield, Yorkshire, UK). Unknown sample cytokine concentrations were automatically calculated by the STarStation software based on a nine-point standard curve (in duplicate) fitted with a four- or five-parameter logistic regression algorithm to allow a wide range of quantification, and *R*^2 ^values were routinely > 0.95. Of the 23 cytokines analysed by using this kit, most had a lower limit of detection that was < 3 pg/mL and an upper limit of detection that was well in excess of 15,000 pg/mL. Intra-assay coefficients of variation were routinely < 15%, indicating a good level of precision. Please refer to Bio-Rad Bulletin 3156 for a detailed summary of the performance characteristics of the Bio-Plex Pro Mouse Cytokine 23-plex panel that we used. The profile of the cytokines analysed was IL-1α, IL-1β, IL-2, IL-3, IL-4, IL-5, IL-6, IL-9, IL-10, IL-12 p40, IL-12 p70, IL-13, IL-17, eotaxin (CCL11), G-CSF, GM-CSF, IFN-γ, KC, MCP-1, MIP-1α, MIP-1β, RANTES and TNF-α.

#### Serum corticosterone concentrations

Offspring serum corticosterone levels were measured using a quantitative competitive ELISA kit (AssayMax Corticosterone ELISA Kit; AssayPro, St. Charles, MO, USA).

### Statistical analysis

All statistics were calculated using SPSS version 14 software (SPSS Inc., Chicago, IL, USA). One-way analysis of variance with the Bonferroni correction was used to analyse all maternal, birth and blastocyst cell number data. All postnatal data were analysed using a multilevel random effects regression model without the Bonferroni correction, which enabled accurate consideration of the hierarchical nature of the data set with between-mother and within-mother variation and incorporation of gestational litter size [16,37]. Thus, differences identified between treatment groups are independent of maternal origin of litter and litter size.

## Abbreviations

BMI: body mass index; BSA: bovine serum albumin; DOHaD: developmental origins of health and disease; ELISA: enzyme-linked immunosorbent assay; ET: endotoxin tolerance; GD: gestational day; GR: glucocorticoid receptor; HPA: hypothalamic-pituitary-adrenal; ICM: inner cell mass; IFN: interferon; IL: interleukin; LPS: lipopolysaccharide; MPSI: maternal periconceptional systemic inflammation; PPAR: peroxisome proliferator-activated receptor; SBP: systolic blood pressure; TE: trophectoderm; TLR: Toll-like receptor; TNF: tumour necrosis factor.

## Authors' contributions

CLW designed the research, performed the experiments and wrote the paper. JLT analysed part of the data and revised the paper. VHP designed the research and revised the paper. TPF designed the research and wrote the paper. All authors read and approved the final manuscript.

## Supplementary Material

Additional file 1 **Figure S1. Maternal serum cytokine concentrations (pg/mL) 3 to 72 hours following treatment with saline or LPS**. Bars: maternal LPS treatment; white: saline control; dark: 10 μg/kg; light: 50 μg/kg; hatched: 150 μg/kg. All values represent means ± SEM. *P ≤ 0.05 compared with control (*n *= 5 to 7 mothers per treatment). The multiplex assay allowed us to detect and quantify the full concentration range of every cytokine at every time point examined, except for G-CSF, KC and IL-6 at three hours postinjection. The data presented here for G-CSF, KC and IL-6 at three hours postinjection are underrepresentative of actual concentrations, particularly for the LPS-treated groups (indicated by ! on graphs). Click here for file

Additional file 2 **Table S1. Embryos flushed from control and LPS-treated mothers at GD 3.5**. Values represent means ± SEM (*n *= 4 to 8 mothers per treatment). Click here for file

Additional file 3 **Figure S2. No difference in growth of female or male offspring from birth to 30 weeks**. Values represent means ± SEM independent of maternal origin and litter size (*n *= 6 mothers per treatment; *n *= 18 male and 18 female offspring per treatment). Click here for file

Additional file 4 **Figure S3. Lifetime mean distance travelled and time spent resting by adult offspring in the open-field behavioural test**. Bars: maternal LPS treatment; white: saline control; dark: 10 μg/kg; light: 50 μg/kg; hatched: 150 μg/kg. Values represent means ± SEM independent of maternal origin and litter size (*n *= 6 mothers per treatment; *n *= 18 male and 18 female offspring per treatment). Click here for file

Additional file 5 **Figure S4. Individual weekly open-field activity of adult male and female offspring**. Values represent means ± SEM. *P ≤ 0.05 vs. control independent of maternal origin and litter size (*n *= 6 mothers per treatment; *n *= 18 male and 18 female offspring per treatment). Click here for file

Additional file 6 **Figure S5. Individual weekly systolic blood pressure (SBP) of adult male and female offspring**. Values represent means ± SEM. *P ≤ 0.05 vs. control independent of maternal origin and litter size (*n *= 6 mothers per treatment; *n *= 18 male and 18 female offspring per treatment). Click here for file

Additional file 7 **Figure S6. Body composition of adult offspring**. **(A) **Organs to body weight (%) of males. **(B) **Fat pad to body weight ratio (%) and BMI of females. **(C) **Organs to body weight ratio (%) of females. **(D) **Liver to body weight ratio (%) in males and females. All values represent means ± SEM. *P ≤ 0.05 vs. control independent of maternal origin and litter size (*n *= 6 mothers per treatment; *n *= 18 male and 18 female offspring per treatment). Click here for file

Additional file 8 **Figure S7. Serum cytokine concentrations (pg/mL) of adult offspring from each maternal treatment group 3.5 hours after LPS challenge**. Values represent means ± SEM. *P ≤ 0.05 vs. control independent of maternal origin and litter size (*n *= 6 mothers per treatment). Maternal treatments: Sal = saline; 10 LPS = 10 μg/kg LPS; 50 LPS = 50 μg/kg LPS; 150 LPS = 150 μg/kg LPS. Click here for file

Additional file 9 **Figure S8. Serum corticosterone concentration (ng/mL) of adult offspring from each maternal treatment**. Values represent means ± SEM (*n *= 6 mothers per treatment). Maternal treatments: Sal = saline; 10 LPS = 10 μg/kg LPS; 50 LPS = 50 μg/kg LPS; 150 LPS = 150 μg/kg LPS. Click here for file
