# Supplementary figures and images for: Mouse maternal systemic inflammation at the zygote stage causes blunted cytokine responsiveness in lipopolysaccharide-challenged adult offspring
Source: BMC Biol. 2011 Jul 19;9:49. doi: 10.1186/1741-7007-9-49 (PMC3152940; doi:10.1186/1741-7007-9-49)

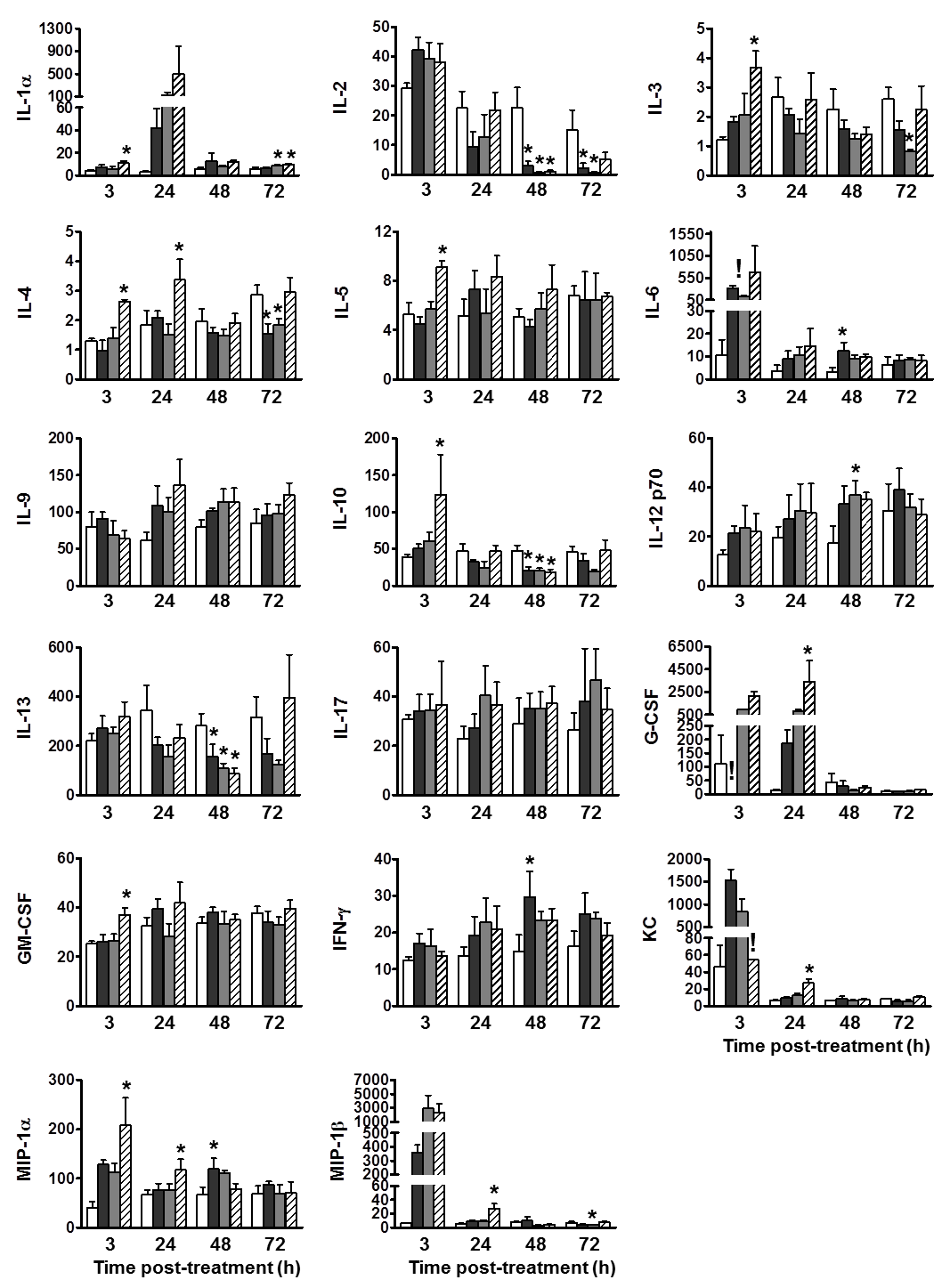

Supplement: Additional file 1 — Figure S1. Maternal serum cytokine concentrations (pg/mL) 3 to 72 hours following treatment with saline or LPS. Bars: maternal LPS treatment; white: saline control; dark: 10 μg/kg; light: 50 μg/kg; hatched: 150 μg/kg. All values represent means ± SEM. *P ≤ 0.05 compared with control (n = 5 to 7 mothers per treatment). The multiplex assay allowed us to detect and quantify the full concentration range of every cytokine at every time point examined, except for G-CSF, KC and IL-6 at three hours postinjection. The data presented here for G-CSF, KC and IL-6 at three hours postinjection are underrepresentative of actual concentrations, particularly for the LPS-treated groups (indicated by ! on graphs). [file 1741-7007-9-49-S1.TIFF]

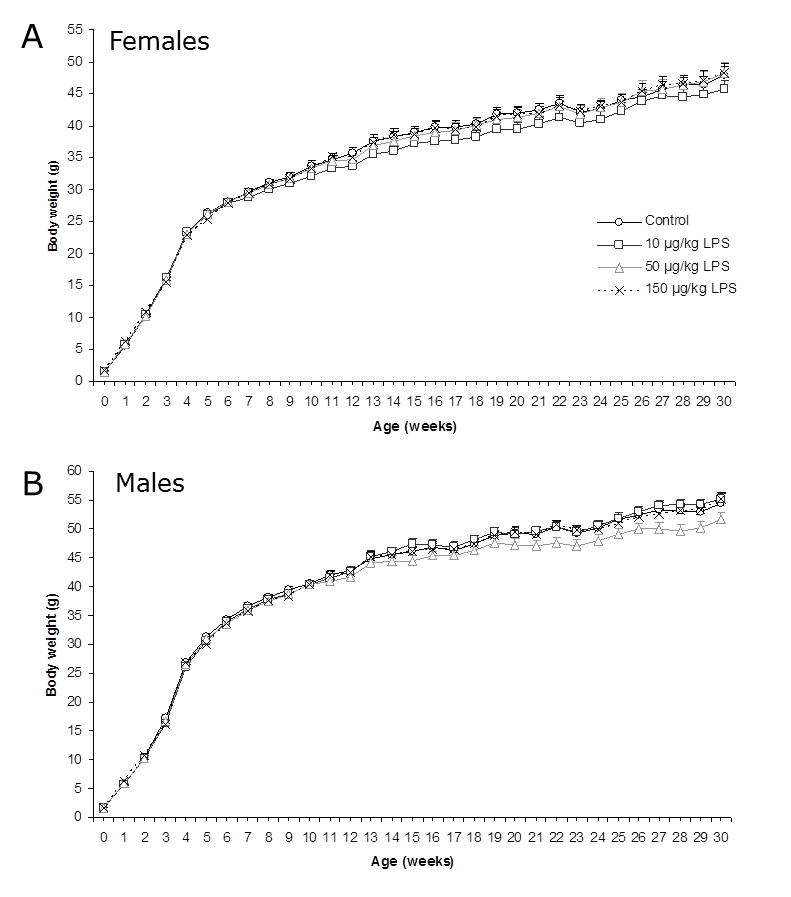

Supplement: Additional file 3 — Figure S2. No difference in growth of female or male offspring from birth to 30 weeks. Values represent means ± SEM independent of maternal origin and litter size (n = 6 mothers per treatment; n = 18 male and 18 female offspring per treatment). [file 1741-7007-9-49-S3.TIFF]

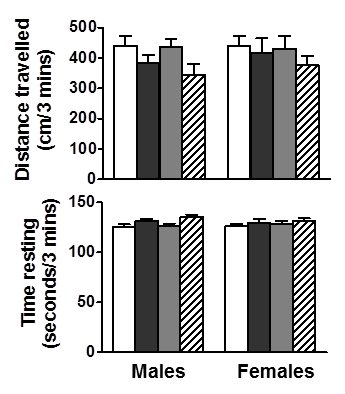

Supplement: Additional file 4 — Figure S3. Lifetime mean distance travelled and time spent resting by adult offspring in the open-field behavioural test. Bars: maternal LPS treatment; white: saline control; dark: 10 μg/kg; light: 50 μg/kg; hatched: 150 μg/kg. Values represent means ± SEM independent of maternal origin and litter size (n = 6 mothers per treatment; n = 18 male and 18 female offspring per treatment). [file 1741-7007-9-49-S4.TIFF]

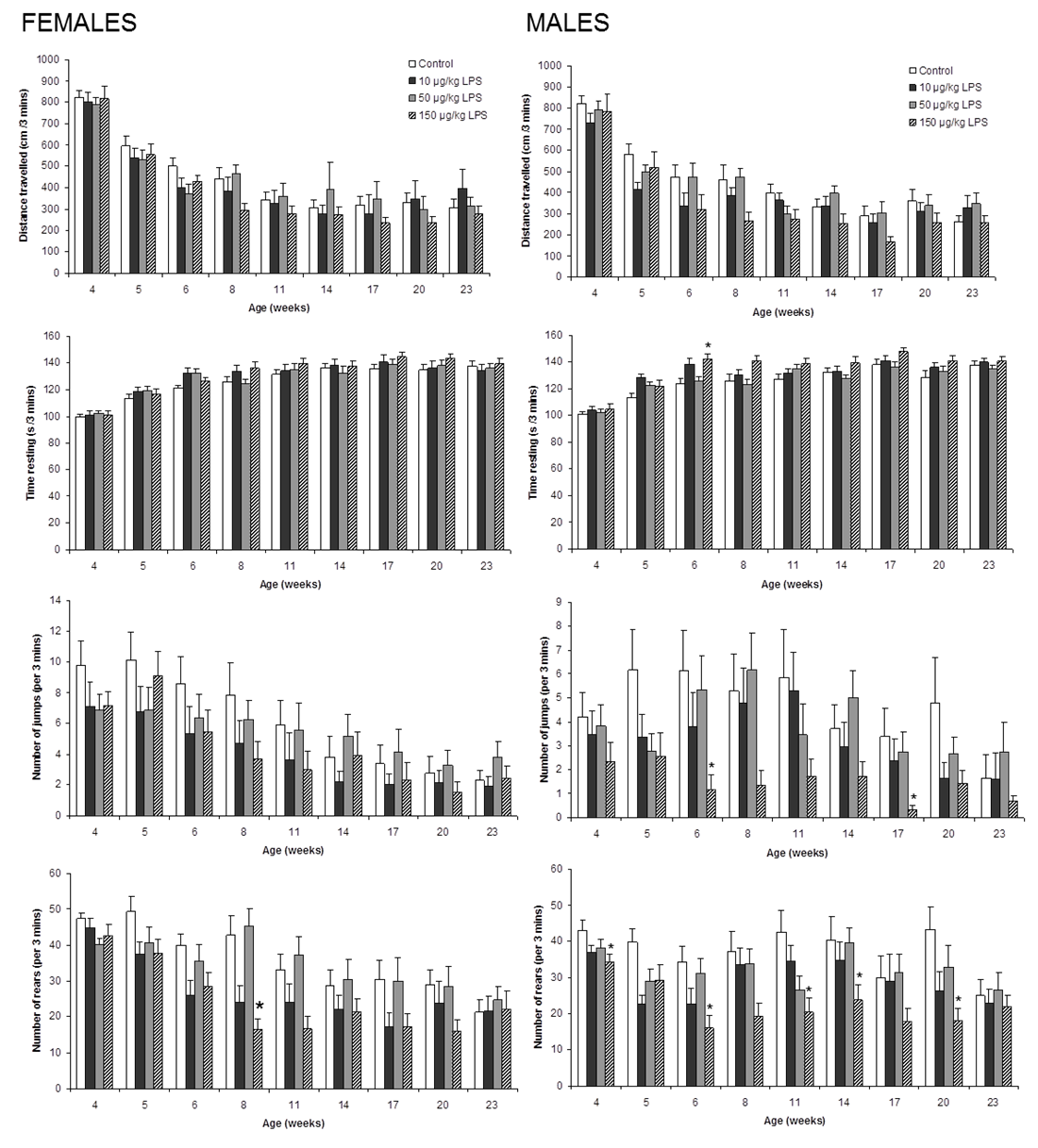

Supplement: Additional file 5 — Figure S4. Individual weekly open-field activity of adult male and female offspring. Values represent means ± SEM. *P ≤ 0.05 vs. control independent of maternal origin and litter size (n = 6 mothers per treatment; n = 18 male and 18 female offspring per treatment). [file 1741-7007-9-49-S5.TIFF]

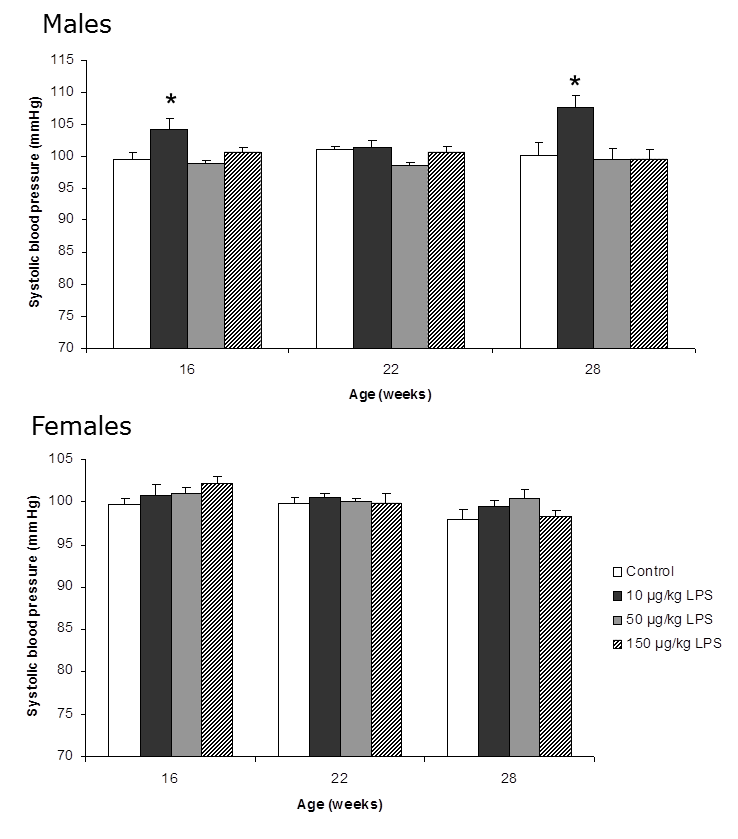

Supplement: Additional file 6 — Figure S5. Individual weekly systolic blood pressure (SBP) of adult male and female offspring. Values represent means ± SEM. *P ≤ 0.05 vs. control independent of maternal origin and litter size (n = 6 mothers per treatment; n = 18 male and 18 female offspring per treatment). [file 1741-7007-9-49-S6.TIFF]

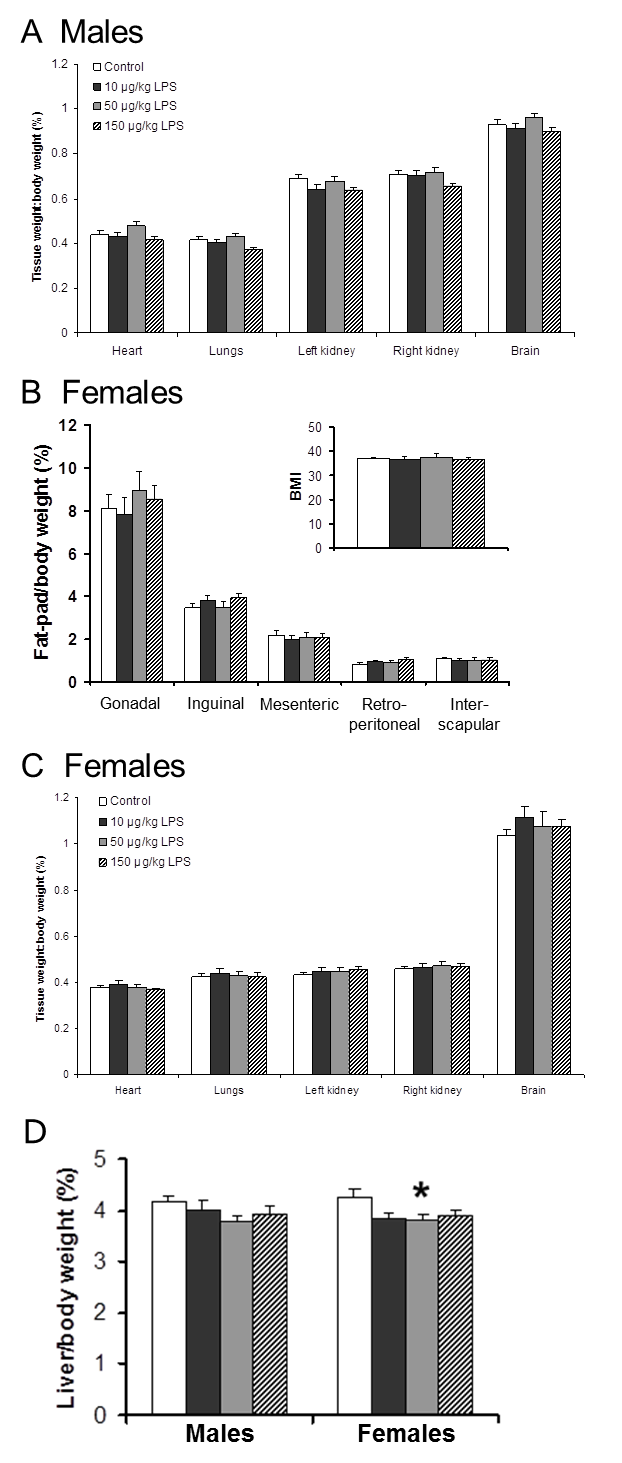

Supplement: Additional file 7 — Figure S6. Body composition of adult offspring. (A) Organs to body weight (%) of males. (B) Fat pad to body weight ratio (%) and BMI of females. (C) Organs to body weight ratio (%) of females. (D) Liver to body weight ratio (%) in males and females. All values represent means ± SEM. *P ≤ 0.05 vs. control independent of maternal origin and litter size (n = 6 mothers per treatment; n = 18 male and 18 female offspring per treatment). [file 1741-7007-9-49-S7.TIFF]

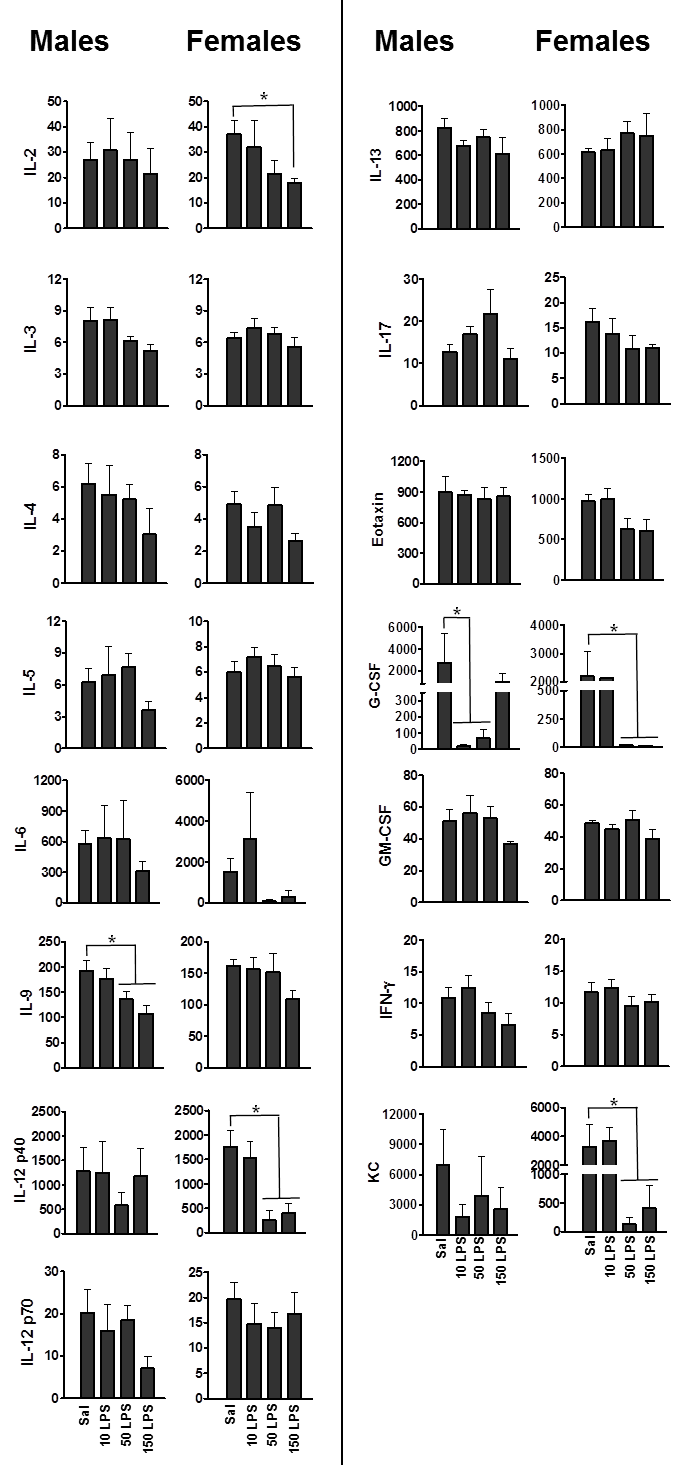

Supplement: Additional file 8 — Figure S7. Serum cytokine concentrations (pg/mL) of adult offspring from each maternal treatment group 3.5 hours after LPS challenge. Values represent means ± SEM. *P ≤ 0.05 vs. control independent of maternal origin and litter size (n = 6 mothers per treatment). Maternal treatments: Sal = saline; 10 LPS = 10 μg/kg LPS; 50 LPS = 50 μg/kg LPS; 150 LPS = 150 μg/kg LPS. [file 1741-7007-9-49-S8.TIFF]

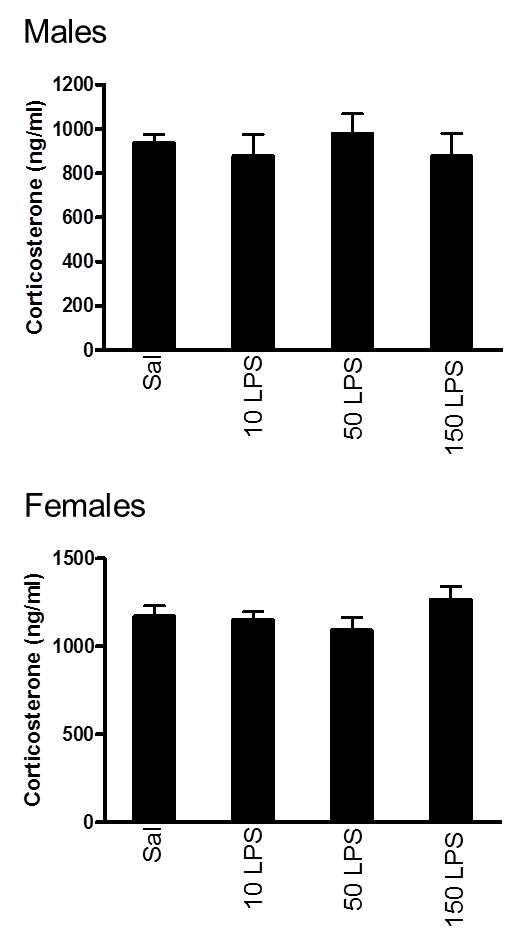

Supplement: Additional file 9 — Figure S8. Serum corticosterone concentration (ng/mL) of adult offspring from each maternal treatment. Values represent means ± SEM (n = 6 mothers per treatment). Maternal treatments: Sal = saline; 10 LPS = 10 μg/kg LPS; 50 LPS = 50 μg/kg LPS; 150 LPS = 150 μg/kg LPS. [file 1741-7007-9-49-S9.TIFF]
