# Supplementary material for: Mouse maternal systemic inflammation at the zygote stage causes blunted cytokine responsiveness in lipopolysaccharide-challenged adult offspring
Source: BMC Biol. 2011 Jul 19;9:49. doi: 10.1186/1741-7007-9-49 (PMC3152940; doi:10.1186/1741-7007-9-49)
Supplement: Additional file 2 — Table S1. Embryos flushed from control and LPS-treated mothers at GD 3.5. Values represent means ± SEM (n = 4 to 8 mothers per treatment). [file 1741-7007-9-49-S2.DOC]

Table S1: Embryos flushed from control- and LPS-treated mothers at GD 3.5 (± S.E.M.; n = 4-8 mothers per treatment).

|  | Maternal Treatment Group (µg/kg LPS) | | | |
| --- | --- | --- | --- | --- |
|  | Control | 10 | 50 | 150 |
| Mean number of embryos flushed | 13.9 ± 1.2 | 15.3 ± 1.0 | 12.1 ± 1.3 | 15.0 ± 0.6 |
| Proportion at blastocyst stage (%) | 64.5 | 87.0 | 65.0 | 62.5 |
